# High-Pressure Processing on Whole and Peeled Potatoes: Influence on Polyphenol Oxidase, Antioxidants, and Glycaemic Indices

**DOI:** 10.3390/foods10102425

**Published:** 2021-10-13

**Authors:** Konstantina Tsikrika, Aine Muldoon, Nora M. O’Brien, Dilip K. Rai

**Affiliations:** 1 Teagasc Food Research Centre Ashtown, Department of Food BioSciences, D15 DY05 Dublin, Ireland; kontsikrika@gmail.com; 2 School of Food and Nutritional Sciences, University College Cork, T12 YN60 Cork, Ireland; 118222208@umail.ucc.ie (A.M.); nob@ucc.ie (N.M.O.)

**Keywords:** high-pressure processing, potatoes, polyphenol oxidase, polyphenols, antioxidant activity

## Abstract

Polyphenol oxidase (PPO) inactivation in five whole and peeled Irish potato cultivars was investigated using high-pressure processing (HPP) at 400 MPa and 600 MPa for 3 min. PPO activity was significantly lower in most of the HPP-treated samples, while the highest PPO inactivation was observed after HPP at 600 MPa. No significant (*p* > 0.05) changes were observed on the total phenolic content and antioxidant activity of all the HPP-treated potatoes. Regarding individual phenolic acids, chlorogenic acid was decreased significantly (*p* < 0.05) in all studied varieties with a concomitant increase (*p* < 0.05) in caffeic and quinic acid. Similarly, ferulic acid was also increased (*p* < 0.05) in all studied varieties after the HPP treatment, while there was a variation in rutin and 4-coumaric acid levels depending on the cultivar and the sample type. Anthocyanins in the coloured whole potato varieties (i.e., Kerr’s Pink and Rooster), tentatively identified as pelargonidin-*O*-ferulorylrutinoside-*O*-hexoside and pelargonidin-*O*-rutinoside-*O*-hexoside, also exhibited significantly (*p* < 0.05) higher levels in the HPP-treated samples as opposed to those untreated. Glycaemic indices of the potatoes treated with HPP did not differ with the corresponding untreated cultivars.

## 1. Introduction

Potato consumption has been associated with beneficial properties such as anti-cancer, hypocholesterolemic, anti-inflammatory, anti-obesity, and anti-diabetic properties in human cell culture, experimental animals, and human clinical studies [1]. Although potatoes have high carbohydrate levels that can result in high glycaemic load (GL), recent reviews present inconsistent effects regarding potato consumption on the risk of type 2 diabetes, obesity, and cardio-metabolic health [2]. In fact, studies have shown that glycaemic index (GI) values decrease in cooled potatoes after cooking [3,4], and are greatly influenced by the cultivar [5]. Furthermore, whole potato consumption can significantly improve the cardioprotective fibre intake [6].

The processing of potatoes prior to their consumption is necessary mostly due to the presence of indigestible ungelatinised starch [7] and the fact that the potato industry has seen promising growth in the demand of minimally processed potatoes, i.e., pre-peeled, fresh cut, or sliced [8]. However, peeling and cutting tubers lead to changes in colour, which could also impact on their sensory properties such as flavour [9]. Polyphenol oxidase (PPO) is the main enzyme causing browning in cut potatoes. PPO catalyses the oxidation of polyphenols that are present in potatoes to quinones, which then undergo further polymerisation into melanin pigments resulting in the aforementioned undesirable changes [10]. Therefore, the inactivation of PPO is essential in order to maintain the quality of potatoes. Although thermal blanching is commonly used to inactivate PPO, its usage has shown a detrimental impact on the nutritional value, physicochemical, and organoleptic characteristics of food [11].

Consumers’ demand for fresh-like products in tandem with enhanced legislation restrictions have led to advancements in food processing technologies that enable a wholesome, fresh product with extended shelf-life [7,12]. In this context, non-thermal processing such as high-pressure processing (HPP) offers a feasible choice against conventional thermal processes [13]. HPP involves applying pressures ranging from 100 to 1000 MPa, commonly at room temperatures and for a short time to packaged foods using water as a medium to transmit pressure [14]. HPP has been applied in foods in order to inhibit the growth of food-borne microorganisms [15,16,17] and enzymes responsible for browning and anaerobic metabolism [18,19,20], while a decreased respiratory activity has also been observed upon the HPP treatment [21].

Previous work of this group has shown that HPP at 600 MPa on whole potatoes exhibited promising results with regard to PPO inactivation while having insignificant impacts on the antioxidant activity, proximate composition, and phytochemical constituents [22,23]. This study further explores the application of two different high-pressure treatments in whole and peeled potatoes of baby potatoes (Maris Piper), two coloured (Kerr’s Pink, Rooster) and two white (Cultra, Maris Piper) varieties that are popular to consumers and widely used by the industry in the island of Ireland. Specifically, the impact of HPP at 400 MPa and 600 MPa was investigated on PPO inactivation, antioxidant activity, polyphenols (including anthocyanins), and their glycaemic indices were determined following the HPP treatments.

## 2. Materials and Methods

### 2.1. Samples

Potatoes of five different cultivars (Kerr’s Pink, Rooster, Maris Piper, Cultra, and Maris Piper baby) and at their commercial maturity were bought from a local supermarket in Dublin, Ireland and were stored in the dark at 4 °C prior to processing.

### 2.2. Chemicals

Hydrochloric acid (HCl), sodium phosphate monobasic, sodium phosphate dibasic, catechol, Folin–Ciocalteu’s phenol reagent, gallic acid, methanol, ethanol, sodium carbonate, formic acid, 6-hydroxy-2,5,7,8-tetramethylchroman-2-carboxylic acid (Trolox), 2,4,6-tripyridyl-striazine, Iron(III) chloride hexahydrate, sodium acetate anhydrous, acetic acid, sodium hydroxide, potassium hydroxide (KOH), guar gum, methanol, 2,2-diphenyl-1-picrylhydrazyl (DPPH), phytochemical standards (quinic acid, chlorogenic acid, caffeic acid, rutin, 4-coumaric acid, ferulic acid), and the enzymes (pepsin, invertase, amyloglucosidase, and pancreatin) were purchased from Merck Ltd. (formerly Sigma Aldrich Ltd., Wicklow, Ireland).

### 2.3. HPP Treatment

Potatoes free of defects were selected, washed, dried, and half of them were peeled. Whole (not-peeled) and peeled potatoes (~1 kg) were packaged separately in polyethylene/polyamide pouches and vacuum-sealed. HPP treatment was performed at 400 MPa and 600 MPa for 3 min with initial temperature at 7.0 °C and reached 10.6 °C (max. temperature reached) (Supplementary Materials Figure S1). Then, 8 kg each of vacuum-packed whole and peeled potatoes of each cultivar were loaded in the 420 Litre Hiperbaric vessel that operated in a horizontal mode on a commercial-scale high pressure process (Hiperbaric 55HT, Miami, FL, USA) that was used located at HPP Tolling (St. Margaret’s, Co., Dublin, Ireland).

### 2.4. Potato PPO Extraction and Activity Assay

The extraction and activity assay of PPO were carried out as previously mentioned [17,23]. Briefly, potato (20 g) was blended with cold phosphate buffer (40 mL; 0.1 M; pH 6.5; 4 °C) for 3 min, and the mixture was centrifuged at 10,000× *g* at 4 °C for 25 min. The supernatant was used to monitor PPO activity. PPO activity assay mixture consisted of phosphate buffer (1.5 mL; 0.1 M; pH 5.5), catechol (1 mL; 0.2 M), and crude PPO extract (0.5 mL). The absorbance was measured at 410 nm for 2 min by a spectrophotometer (UV-1700, Shimadzu, Nanjing, China). The residual activity (RA) of PPO was calculated, using the following Equation (1):

RA (%) = (A_t_/A_0_) × 100 (1)

where A_t_ and A_0_ are PPO activity after and before the treatment, respectively.

### 2.5. Extraction of Phytochemicals

The extraction of phytochemicals was conducted following previously published literature [17,23]. Potato samples were cut into cubes and frozen overnight on silver-foil trays. Freeze drying of the frozen cubes were performed in a Cuddon freeze-drier, model FD80 (Cuddon Freeze Dry, Blenheim, New Zealand) at a temperature of −54 °C and a pressure of 0.064 mbar for 72 h followed by grinding into powders. The extraction of phytochemicals from the lyophilised whole and peeled potatoes was carried out overnight in 80% methanol containing 0.01% formic acid (1:5 *w*/*v*) at 4 °C. This was followed by sonication at 30 °C for 30 min and then centrifuging at 10,000× *g* for 30 min. The extraction process was repeated on the residue. The supernatants were pooled and filtered through 0.45 µm syringe filters.

### 2.6. Total Phenolic Content

The total phenolic content (TPC) of whole and peeled potatoes was assessed using the Folin–Ciocalteu reagent (FCR) method [24] adapted to a microplate reader (FLUOstar Omega Microplate Reader, BMG Labtech GmbH, Offenburg, Germany) as previously described in [17,23]. Potato extract (100 µL) was added to a solution containing FCR (100 µL), sodium carbonate (100 µL; 20% *w*/*v*), and methanol (100 µL). The sample–reagent mixture was allowed to react in the dark for 20 min, and then, it was centrifuged at 13,000 rpm for 3 min. The absorbance was monitored at 735 using gallic acid as a standard, while methanol was used as a blank. The results were calculated as µg of gallic acid equivalent per g of the extract’s dry weight (µg GAE/g dw).

### 2.7. Antioxidant Activity (AOA)

#### 2.7.1. Ferric-Reducing Antioxidant Power (FRAP)

The FRAP assay was carried out following an amended method of Benzie and Strain (1996) [25] by Stratil et al. (2006) [26] and Ou et al. (2002) [27]. The FRAP solution contained acetate buffer (100 mL; 0.3 M; pH 3.6), ferrous chloride hexahydrate (10 mL; 0.01 M) and TPTZ (10 mL; 0.01 M in 0.04 M HCl). Potato extract (20 μL) was added to FRAP solution (180 μL) in the microplate well, and the mixture was kept at 37 °C for 40 min before reading the absorbance at 593 nm. Trolox and methanol were used as standard and blank, respectively. The results were reported as µg of Trolox equivalent per g of the extract’s dry weight (µg TE/g dw).

#### 2.7.2. DPPH Radical Scavenging Capacity

The DPPH radical scavenging capacity of HPP treated and untreated peeled and whole potatoes was assayed as described in Goupy et al. (1999) [28]. A stock solution of DPPH (11.9 mg) and methanol (50 mL) was prepared followed by further a 1:5 dilution of the stock solution for the assay. The diluted DPPH solution (100 μL) was mixed with potato extract (100 μL) and kept in the dark for 30 min. Then, the absorbance was measured at 515 nm in the microplate reader, while Trolox and methanol were used as standard and blank, respectively. The results were reported as µg of Trolox equivalent per g of the extract’s dry weight (µg TE/g dw).

### 2.8. Liquid Chromatography-Mass Spectrometry Analysis

The identification of polyphenols was performed by HPLC-QToF mass spectrometry [23,29] and their quantification by ultra-high performance liquid chromatography coupled to a tandem quadrupole mass spectrometer (UPLC-TQD, Waters Corp., Milford, MA, USA). In addition, two anthocyanins, namely pelargonidin-*O*-ferulorylrutinoside-*O*-hexoside and pelargonidin-*O*-rutinoside-*O*-hexoside, were tentatively identified based on their molecular mass and fragmentation pattern [30]. The separation of the natural compounds in the potato extract was performed on a Waters Acquity UPLC HSS T3 column (100 × 2.1 mm, 1.8 µm). The binary solvents constituted water containing 0.1% formic acid and acetonitrile containing 0.1% formic acid at 0.5 mL/min [31,32]. A multiple reaction monitoring (MRM) mode analysing at least two transitions per compound was employed to detect and quantify the phenolic compounds in the UPLC-TQD. IntelliStart^TM^ software (Masslynx 4.1, Waters Corp., Milford, MA, USA) was used in order to optimise the cone voltages and collision energies for each MRM transition. As for the two tentative anthocyanins, MRM transitions were obtained from Kim et al. 2018 [30]. Quadruplicate extracts were used for the analyses, while the target compounds were quantified using standard calibration curves of concentrations ranging from 10–25 µg/mL. The two anthocyanins were quantified as rutin equivalents. The results were reported as µg compound per g of extract in dry weight (µg/g dw).

### 2.9. In Vitro Determination of Glycaemic Index (GI)

#### 2.9.1. In Vitro Digestion

The enzyme mixture (0.5 g pepsin, 0.5 g guar gum, 100 mL 0.05 mol/L HCl) was prepared, and the reaction mixture was maintained at room temperature. Then, a potato sample (1 g) was added to pepsin–guar gum mixture (10 mL) followed by incubation in a shaking water bath at 37 °C for 30 min. Five glass balls (0.5 cm diameter) were mixed with sodium acetate (10 mL; 0.25 mol/L), along with the second enzyme mixture (5 mL), which contained invertase (2000 units/mL), amyloglucosidase, and pancreatin. Then, samples were incubated in a 37 °C water bath, and after 20 min and 120 min, an aliquot of 0.2 mL was taken out and mixed with 20 mL of 66% ethanol for glucose analysis (these were the samples, which were used to calculate the rapidly available glucose (RAG) and slowly available glucose (SAG). The remaining mixture was vortexed and then incubated for 30 min at 100 °C. The mixture was cooled for 20 min in ice bath; then, we added KOH (10 mL; 7 mol/L) followed by an incubation at 0 °C in a shaking ice bath for 30 min. The mixture was vortexed, and 1 mL of solution mixture was transferred into a heat-resistant beaker, which contained acetic acid (10 mL; 0.5 mol/L) and amyloglucosidase (0.2 mL). Then, this mixture was incubated for 30 min at 70 °C in a shaking water bath. The mixture was taken out from the water bath, after which 40 mL of distilled water was added, and the mixture was vortexed. This was the sample used to determine total glucose (TG), which was performed immediately [33].

#### 2.9.2. Glucose Analysis

Glucose was determined using the Megazyme D-glucose assay kit (GOPOD-format). In glass heat-resistant tubes, 3 mL of GOPOD reagent was mixed with 0.1 mL of sample solution (RAG, SAG, TG, and FG (free glucose)). Then, the sample-reagent mixture was incubated for 20 min at 50 °C, and the absorbance was measured at 510 nm on the spectrophotometer.

Three independent experiments were carried out in all studies. Each sample was tested in duplicate (n = 6).

Equations (2)–(6) as described by Englyst et al. (2000) [33] were used to calculate the GI, GL, total starch (TS), RAG, and SAG:

(2) $${\mathrm{RAG}}_{\mathrm{rel}}=\frac{\mathrm{RAG}\times 100}{\mathrm{TG}}$$

(3) $${\mathrm{SAG}}_{\mathrm{rel}}=\frac{\mathrm{SAG}\times 100}{\mathrm{TG}}$$

(4) TS=0.9×(TG−FG)

(5) $$\mathrm{GI}=17.7+77.9\frac{\mathrm{RAG}}{\mathrm{TS}+2\mathrm{FG}}$$

(6) $$\mathrm{GL}=\mathrm{GI}\frac{\mathrm{TS}+2\mathrm{FG}}{100}.$$

### 2.10. Statistical Analysis

Experiments were performed in duplicates, and all analyses (except for GI) were repeated four times (n = 8). Results are reported as means ± standard deviation (SD). One-way ANOVA followed by a Tukey’s post hoc test was performed for the statistical analysis of PPO RA before/after treatment, while a Games–Howell post hoc test was used for the rest of the data. SPSS Statistics 26 (IBM-Armonk, New York, NY, USA) was used for all the statistical analyses. The significance level was set at *p* < 0.05.

## 3. Results

### 3.1. PPO Inactivation

**Figure 1:**
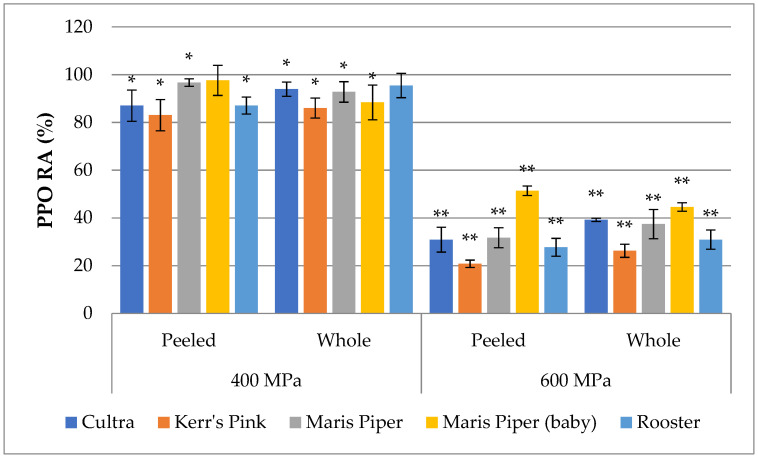
Residual activity (%) of PPO from whole and peeled potatoes upon HPP treatment at 400 MPa and 600 MPa. ‘*’ indicates statistically significant (*p* < 0.05) difference from the control. ‘**’ indicates statistically significant difference (*p* < 0.05) within the same cultivar and sample type (peeled/whole).

The effect of HPP treatments on the activity of PPO expressed as residual activity (RA) is shown in Figure 1. Although HPP at 400 MPa and 600 MPa for 3 min each decreased the PPO activity significantly (*p* < 0.05) in both the peeled and whole potatoes, the HPP at 600 MPa was more effective against PPO, where the RA ranged from approximately 21% to 51% in peeled and 23% to 53% in whole potatoes. As with the HPP at 400 MPa, the RA of the PPO varied from approximately 83% to 93% in peeled and 86% to 97% in whole samples of the studied cultivars, while no significant changes (*p* > 0.05) were observed in the peeled baby Maris Piper and whole Rooster samples as compared to the controls. In general, a higher PPO activity was found in the whole than in the peeled potatoes irrespective of different pressure (400 MPa and 600 MPa) treatments, which was also noted by Thygesen et al. (1995) [34]. The PPO RA values of whole potatoes are similar to our previous study where it was found to vary from 31% to 51% after HPP at 600 MPa [23]. Similarly, a 30% inactivation of PPO by HPP at 600 MPa has been reported in carrot juice [19], while HPP at 400 MPa and 600 MPa decreased PPO by 11% and 30%, respectively in strawberry puree [35]. A significant (*p* < 0.05) reduction in PPO RA was also observed in a commercial PPO isolated from mushroom under HPP at 600 MPa for 5 min [36], and in Peruvian carrot and cocoyam puree, cubes, and extract after HPP treatment at 600 MPa for 5 or 30 min. On the contrary, PPO activity in HPP-treated sweet potato was much higher, reaching even four times higher RA than the controls, while a higher PPO activity was also observed in HPP-treated avocado slices than in those untreated [21]. These findings indicate the importance and relevance of the food matrix in PPO inactivation by HPP.

HPP-induced enzyme inactivation is a multifaceted phenomenon that has not been fully determined yet. Potential mechanisms involve the formation and/or disruption of various intramolecular interactions, hydration of charged groups, interference of bound water, the stabilisation of hydrogen bonds, as well as alterations in the native structure of enzymes by folding and/or unfolding [36]. HPP may lead to reversible or irreversible and partial or complete unfolding of the native structure of the enzyme, resulting in a change in enzyme activity, as its specificity is very much associated to its active sites in the protein structure [37]. From a thermodynamic perspective, HPP may influence enzyme-catalysed reactions by modifying the equilibrium and the rate constants [38]. One or a combination of these factors might have contributed to the observed loss of PPO activity in the current study.

### 3.2. Total Phenolic Content

**Figure 2:**
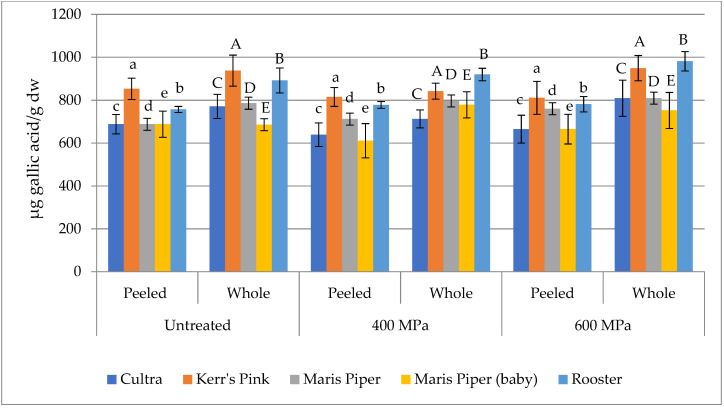
Total phenolic content (TPC) as expressed as μg gallic acid equivalent (GAE)/g dry weight in untreated and HPP treated peeled and whole potatoes. Different letters above bars indicate a statistically significant (*p* < 0.05) difference within the same cultivar and sample type (peeled and whole).

Figure 2 shows the total phenolic content (TPC) of peeled and whole potatoes of the studied cultivars before and after HPP treatment. TPC in untreated potatoes was found to range from 568 to 853 μg GAE/g dw in peeled, while it was higher in whole samples (686 to 938 μg GAE/g dw), which is consistent with the literature [39]. As shown in Figure 2, the impact of HPP on TPC of peeled and whole potatoes exhibits a variation, mainly depending on the cultivar. Nevertheless, the changes are not significant (*p* > 0.05). These results contradict previous findings where TPC levels were significantly (*p* < 0.05) higher in whole potatoes after HPP at 600 MPa than in those untreated [23]. This difference may be due to the fact that the levels of phenolic compounds in the potatoes as well as their stability and consequently the effect of a processing method on them are highly dependent on several factors such as genetic (cultivar), environmental, agronomic, the stage of ripeness, and the post-harvest handling and storage [40,41]. No significant changes in TPC have also been reported in HPP treated mango nectars [42], litchi juice [43], and acai juice [44] with respect to the controls. On the contrary, a significant increase was observed in TPC in pumpkin slices after HPP at 450 MPa for 15 min and 550 MPa for 10 min [16] and in HPP-treated pomegranate juice [33] than in the corresponding untreated samples, which was associated with a higher cell permeability, as a consequence of the disruption of the cell walls and the cell membrane hydrophobic bonds, and thereby leading to mass release of matrix-bound phenolic compounds.

### 3.3. Antioxidant Activity

**Figure 3:**
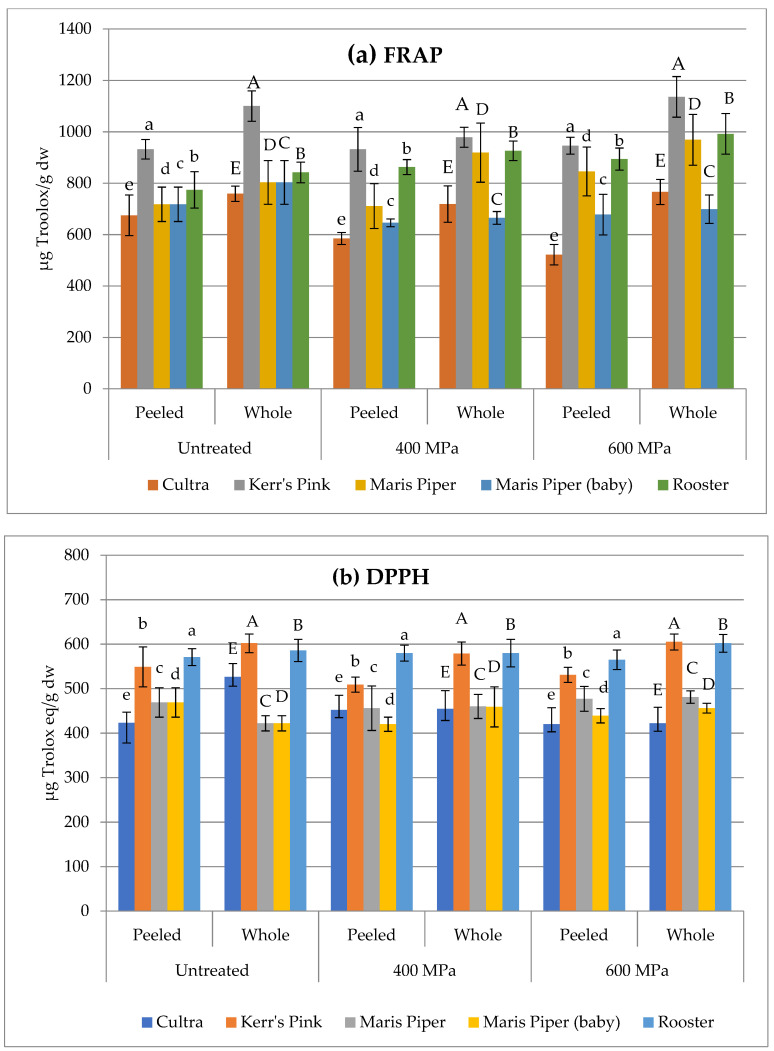
DPPH radical scavenging capacity (**a**) and FRAP (**b**) as expressed as μg Trolox equivalent (TE)/g dry weight in peeled and whole, untreated, and HPP-treated potatoes. Different letters above bars indicate a statistically significant (*p* < 0.05) difference within the same cultivar and sample type (peeled and whole).

The antioxidant activity (AOA) of untreated and HPP treated, peeled and whole potato samples as examined by FRAP assay and DPPH radical scavenging capacity is shown in Figure 3a,b, respectively. Similar to the TPC results, AOA assayed by either DPPH or FRAP showed a variation after the HPP treatments, depending on the treatment pressure, cultivar, and the type (peeled or whole) of the sample. Nevertheless, the changes in AOA were insignificant (*p* > 0.05), which was also observed in our previous work following 600 MPa treatment of whole potatoes [22]. Studies on other food products, namely aronia berry puree [45], mango nectars [42], and purple sweet potato nectars [46], have also shown no significant (*p* > 0.05) changes in their AOA following HPP treatments ranging from 400 to 600 MPa and for 1–10 min. The authors of these studies linked their findings to unaltered levels of the TPC, which can also explain the results of the current study. Conversely, AOA was slightly but significantly (*p* < 0.05) increased in smoothies [14] and in pumpkin [16] upon HPP, whereas a significant (*p* < 0.05) decrease was found in *Aloe Vera* gel after HPP at 150, 250, 350, 450, and 550 MPa for 5 min [47]. These findings indicate that the AOA in HPP-treated products is dependent on the state of the substrate studied, where the AOAs of those that are in liquid or semi-liquid forms can be altered by the HPP treatment.

### 3.4. Effect of HPP on Polyphenols

Table 1 and Table 2 show the effect of HPP on individual polyphenols in whole and peeled potatoes. Chlorogenic acid was the most abundant polyphenol in all of the studied samples, and it varied from approximately 36 to 97 μg/g dw and 35 to 51 μg/g dw in whole and peeled samples, respectively as observed by other authors [48]. A significant (*p* < 0.05) decrease in chlorogenic acid both in peeled and whole potatoes was noted after the HPP treatments. A simultaneous increase (*p* < 0.05) of chlorogenic acid’s constituent phenolic acids, i.e., caffeic acid and quinic acid, was also observed, which suggests that HPP induces degradation. Similarly, levels of rutin, which is a rutinoside of quercetin, were slightly reduced in the HPP-treated peeled samples as opposed to the untreated peeled samples, although the changes were not significant (*p* > 0.05). A significant (*p* < 0.05) increase was found in the ferulic acid content in most of the HPP-treated samples, and in particular, Maris Piper cultivars showed almost six times higher levels as compared to those untreated, suggesting that ferulic acid occurs possibly also in bound forms in these varieties. It is known that hydroxycinnamic acids (i.e., ferulic acid, 4-coumaric acid, chlorogenic acid, isochlorogenic acid, caffeic acid, and sinapic acid) and some flavonoids ((-)epicatechin, quercetin, and rutin) occur in free and bound forms, and that HPP can alter their concentrations [49].

Two anthocyanins, namely pelargonidin-*O*-feruloylrutinoside-*O*-hexoside and pelargonidin-*O*-coumarylrutinoside-*O*-hexoside, have been tentatively identified in whole Kerr’s Pink and Rooster samples based on mass spectral data and literature [50], and their levels were measured. Both HPP treatments led to a significant (*p* < 0.05) increase of these two anthocyanins in these coloured cultivars. PPO inactivation has been associated with the stability of the anthocyanins in HPP-treated (800 MPa, 18–22 °C for 15 min) red raspberry and strawberry fruits [51], which is consistent with the findings of this study where PPO RA was very low in Kerr’s Pink and Rooster potatoes after the HPP at 600 MPa. As discussed earlier in Section 3.2 and Section 3.3, HPP might cause cell-wall damage resulting in the release of phytochemicals including anthocyanins into the extracellular environment.

### 3.5. Glycaemic Index (GI) of Potato Cultivars

The amount of total glucose that is released rapidly (RAG) is one of the key determinants of the GI of the food. All the untreated potato cultivars had a high GI value apart from Maris Piper, which had a medium value. According to the literature, the GI of British potatoes varies from 56 to 94 [5], whilst a variance of 53 to 103 was seen between Australian potato varieties [52]. It could be hypothesized that the variation in the GI of potato cultivars is due to structural differences of the starch in the tubers. For instance, the in vitro and in vivo studies investigating the GI of rice had shown that the cultivars with high levels of resistant starch and amylose decreased the GI [53,54].

In the present study, statistically significant decreases were observed in the GI values of both coloured potato cultivars (Roosters and Kerr’s Pink) and Maris Piper (baby) following 600 MPa treatment. For the rest of the samples, there were no statistically significant (*p* > 0.05) differences in the GI values irrespective of HPP treatment or not (Table 3).

Previously published literature suggested that the HPP treatments might result in a decrease in GI [55,56]. However, in both these studies, they used in vivo methodologies as well as ready-to-eat foods with no further processing after the HPP, whilst in the present study, potatoes were cooked following the HPP before the in vitro digestion was carried out. Nasehi et al. (2012) [57] suggested that owing to its high water content, potato starch is more resistant to pressure than the starch present in rice, corn, or tapioca, which may explain the unaltered GI values in some of the potato cultivars studied here. The application of cycles of HPP and longer periods of HPP, or a long continuous HPP treatment instead of the single treatment for three minutes may cause the desired changes in the starch structure of the potato. This can subsequently impede the release of glucose as well as the breakdown of the starch and thereby reduce the GI and GL of the potato [58].

## 4. Conclusions

HPP at 600 MPa for 3 min is efficient in inhibiting PPO activity by as much as 79% in peeled potatoes. The HPP treatments do not alter total phenolic content and antioxidant activities of whole and peeled potatoes irrespective of the strength of the applied pressures. However, the HPP treatments can induce the degradation of (poly)phenolic acid conjugates such as chlorogenic acid, which is an ester of caffeic acid and quinic acid. Chlorogenic acid is the most abundant polyphenol in potatoes, which is significantly decreased upon HPP treatments with concurrent increases in caffeic acid and quinic acid. On the other hand, HPP led to an increase in ferulic acid, which is most commonly found bound to cell walls in whole and peeled samples. Similarly, the anthocyanins pelargonidin-*O*-ferulorylrutinoside-*O*-hexoside and pelargonidin-*O*-rutinoside-*O*-hexoside in the HPP-treated whole samples of the coloured potato varieties increased significantly, implying that the high hydrostatic pressure disrupts the cell walls, enabling the release of bound (poly)phenols. Despite the alterations of individual (poly)phenols by the impact of HPP, the overall antioxidant capacity was not affected. No significant effect was observed on the carbohydrate parameters, i.e., glycaemic index and glycaemic load of the HPP-treated potato cultivars. HPP is a promising technology in potato processing that can be applied to inhibit browning while maintaining nutritional and functional qualities. Further studies on the combination of HPP with other novel processing technology(ies) or natural antioxidant(s) are necessary to establish the validity as alternate to thermal and/or chemical processing of minimally processed potatoes, which is beyond the scope of the current study.

## Figures and Tables

**Table 1:** Means ± SD (n = 8) of the phytochemical content (µg/g dry weight) in untreated and HPP-treated whole potatoes.

Cultivar	Treatment	Polyphenols						Anthocyanins (Rutin Equivalent)	
		Ferulic Acid	Chlorogenic Acid	Caffeic Acid	Quinic Acid	4-Coumaric Acid	Rutin	Pelargonidin-*O*-Feruloylrutinoside-*O*-Hexoside	Pelargonidin-*O*-Rutinoside-*O*-Hexoside
Cultra	Untreated	0.4 ± 0.02 ^b^	35.9 ± 1.1 ^ab^	18.0 ± 1.6 ^b^	71.3 ± 2.9 ^ab^	3.6 ± 0.5 ^b^	0.66 ± 0.1 ^b^	ND	ND
	400 MPa	3.1 ± 0.7 ^a^	37.5 ± 3.1 ^ab^	23.5 ± 4.5 ^a^	99.0 ± 1.8 ^a^	10.1 ± 1.5 ^a^	0.85 ± 0.2 ^b^	ND	ND
	600 MPa	2.7 ± 0.3 ^a^	34.8 ± 2.1 ^ab^	19.7 ± 4.2 ^a^	86.2 ± 2.7 ^b^	9.0 ± 0.5 ^a^	1.85 ± 0.2 ^a^	ND	ND
Kerr’s Pink	Untreated	0.5 ± 0.2 ^b^	54.0 ± 1.3 ^a^	18.3 ± 2.5 ^ab^	32.5 ± 3.7 ^b^	2.4 ± 0.8 ^ab^	0.5 ± 0.1 ^a^	1.8 ± 0.3 ^b^	2.0 ± 0.2 ^a^
	400 MPa	0.6 ± 0.2 ^b^	50.4 ± 1.8 ^b^	48.3 ± 1.3 ^b^	75.2 ± 3.5 ^a^	23.6 ± 1.1 ^a^	0.5 ± 0.1 ^a^	3.5 ± 1.0 ^a^	2.7 ± 0.6 ^a^
	600 MPa	0.9 ± 0.3 ^b^	47.5 ± 3.3 ^b^	60.4 ± 1.9 ^a^	72.9 ± 3.2 ^a^	20.5 ± 0.1 ^b^	0.6 ± 0.1 ^a^	3.0 ± 0.3 ^a^	2.5 ± 0.5 ^a^
Maris Piper	Untreated	0.4 ± 0.1 ^b^	55.3 ± 4.1 ^a^	28.3 ± 3.6 ^ab^	48.8 ± 1.7 ^ab^	10.4 ± 0.8 ^b^	5.1 ± 0.3 ^a^	ND	ND
	400 MPa	3.4 ± 0.2 ^a^	38.3 ± 9.80 ^ab^	56.8 ± 7.9 ^b^	57.0 ± 5.4 ^b^	15.2 ± 0.6 ^a^	5.6 ± 0.2 ^a^	ND	ND
	600 MPa	3.2 ± 0.1 ^a^	38.9 ± 4.8 ^ab^	68.2 ± 8.9 ^a^	88.8 ± 4.6 ^a^	15.5 ± 0.6 ^a^	5.4 ± 0.2 ^a^	ND	ND
Maris Piper (baby)	Untreated	0.4 ± 0.1 ^b^	55.3 ± 4.1 ^a^	28.3 ± 3.6 ^ab^	48.8 ± 1.7 ^ab^	10.4 ± 0.8 ^ab^	5.1 ± 0.3 ^a^	ND	ND
	400 MPa	3.3 ± 0.1 ^a^	43.9 ± 3.8 ^b^	80.1 ± 3.8 ^a^	77.9 ± 2.2 ^b^	17.4 ± 0.8 ^b^	4.9 ± 0.4 ^a^	ND	ND
	600 MPa	3.6 ± 0.3 ^a^	44.6 ± 3.6 ^b^	77.3 ± 4.0 ^b^	107 ± 12 ^a^	19.6 ± 1.2 ^a^	4.4 ± 0.7 ^a^	ND	ND
Rooster	Untreated	0.5 ± 0.01 ^b^	60.8 ± 2.0 ^a^	16.3 ± 2.0 ^ab^	50.1 ± 2.9 ^ab^	9.4 ± 1.1 ^ab^	1.8 ± 0.4 ^a^	9.9 ± 0.5 ^b^	6.5 ± 1.7 ^b^
	400 MPa	0.4 ± 0.03 ^b^	36.6 ± 5.1 ^ab^	21.3 ± 4.3 ^b^	86.8 ± 7.0 ^a^	24.5 ± 3.6 ^a^	2.3 ± 0.4 ^a^	14.7 ± 3.2 ^a^	11.1 ± 5.4 ^a^
	600 MPa	0.4 ± 0.03 ^b^	30.7 ± 2.6 ^ab^	24.5 ± 0.7 ^a^	76.5 ± 8.2 ^b^	16.1 ± 2.3 ^b^	2.1 ± 0.2 ^a^	18.2 ± 3.5 ^a^	11.8 ± 3.0 ^a^

Values with different superscript letters within the same column and cultivar are significantly different (*p* < 0.05).

**Table 2:** Means ± SD (n = 8) of the phytochemical content (µg/g dry weight) untreated and HPP-treated peeled potatoes.

Cultivar	Treatment	Polyphenols					
		Ferulic Acid	Chlorogenic Acid	Caffeic Acid	Quinic Acid	4-Coumaric Acid	Rutin
Cultra	Untreated	ND	35.2 ± 2.5 ^a^	5.7 ± 1.1 ^a^	32.9 ± 2.6 ^ab^	2.3 ± 0.4 ^a^	0.5 ± 0.1 ^a^
	400 MPa	ND	19.5 ± 1.0 ^b^	5.9 ± 1.1 ^a^	58.3 ± 4.5 ^b^	1.9 ± 0.4 ^a^	0.3 ± 0.1 ^b^
	600 MPa	ND	2.5 ± 0.5 ^ab^	0.4 ± 0.1 ^b^	111.2 ± 6.9 ^a^	ND	0.3 ± 0.1 ^b^
Kerr’s Pink	Untreated	0.6 ± 0.1 ^a^	47.5 ± 8.3 ^a^	6.3 ± 1.0 ^a^	36.1 ± 3.1 ^ab^	1.9 ± 0.3 ^a^	0.6 ± 0.1 ^a^
	400 MPa	0.5 ± 0.05 ^a^	26.4 ± 4.4 ^b^	6.9 ± 1.7 ^a^	48.7 ± 2.3 ^b^	1.3 ± 0.2 ^b^	0.2 ± 0.1 ^b^
	600 MPa	0.5 ± 0.05 ^a^	1.1 ± 0.2 ^ab^	0.2 ± 0.05 ^b^	57.9 ± 2.5 ^a^	1.5 ± 0.1 ^b^	0.2 ± 0.1 ^b^
Maris Piper	Untreated	0.6 ± 0.1 ^b^	38.1 ± 4.9 ^a^	34.1 ± 2.7 ^ab^	27.1 ± 3.2 ^ab^	1.4 ± 0.1 ^a^	0.7 ± 0.1 ^a^
	400 MPa	1.6 ± 0.5 ^a^	4.1 ± 0.5 ^b^	39.5 ± 1.3 ^b^	85.2 ± 1.5 ^b^	1.2 ± 0.1 ^b^	0.3 ± 0.2 ^b^
	600 MPa	2.3 ± 0.4 ^a^	4.2 ± 0.2 ^b^	43.5 ± 1.5 ^a^	138 ± 7.9 ^a^	1.0 ± 0.2 ^b^	0.3 ± 0.1 ^b^
Maris Piper (baby)	Untreated	0.5 ± 0.1 ^b^	38.5 ± 3.7 ^a^	31.4 ± 1.6 ^a^	21.5 ± 6.3 ^ab^	1.4 ± 0.3 ^a^	0.8 ± 0.2 ^a^
	400 MPa	2.3 ± 1.0 ^a^	3.2 ± 0.6 ^b^	26.7 ± 0.7 ^b^	57.2 ± 1.8 ^b^	1.1 ± 0.2 ^b^	0.6 ± 0.1 ^a^
	600 MPa	2.4 ± 0.8 ^a^	2.5 ± 0.2 ^b^	28.9 ± 1.4 ^a^	116 ± 11 ^a^	1.2 ± 0.2 ^b^	0.7 ± 0.1 ^a^
Rooster	Untreated	0.3 ± 0.05 ^b^	50.9 ± 4.7 ^a^	14.1 ± 0.8 ^a^	36.1 ± 2.9 ^ab^	2.5 ± 0.5 ^a^	0.8 ± 0.2 ^a^
	400 MPa	2.0 ± 0.1 ^a^	10.6 ± 0.5 ^b^	5.6 ± 0.4 ^b^	63.2 ± 4.3 ^b^	2.2 ± 0.3 ^a^	0.4 ± 0.1 ^b^
	600 MPa	2.5 ± 0.1 ^a^	5.5 ± 0.7 ^ab^	3.0 ± 0.2 ^ab^	77.1 ± 1.7 ^a^	2.1 ± 0.4 ^a^	0.4 ± 0.2 ^b^

Values with different superscript letters within the same column and cultivar are significantly different (*p* < 0.05).

**Table 3:** Carbohydrate parameters, glycaemic index and glycaemic load of untreated and HPP-treated potatoes.

Cultivars	Treatment	Rapidly Available Glucose (g/100 g)	Total Glucose (g/100 g)	Glycaemic Index	Glycaemic Load
Cultra	Untreated	14.38 ± 1.02 ^a^	19.31 ± 0.77 ^a^	79.10 ± 6.79 ^a^	14.09 ± 1.07 ^a^
	400 MPa	11.73 ± 0.39 ^b^	18.92 ± 2.27 ^a^	68.36 ± 10.52 ^a^	11.73 ± 0.72 ^b^
	600 MPa	14.93 ± 0.75 ^a^	20.18 ± 3.43 ^a^	80.97 ± 7.72 ^a^	14.92 ± 1.07 ^a^
Kerr’s Pink	Untreated	10.77 ± 0.18 ^a^	11.39 ± 0.51 ^a^	97.79 ± 3.42 ^a^	10.24 ± 0.17 ^a^
	400 MPa	9.23 ± 1.29 ^a^	12.06 ± 4.15 ^a^	85.00 ± 12.05 ^a^	9.16 ± 1.69 ^a^
	600 MPa	9.37 ± 1.95 ^a^	17.33 ± 2.10 ^b^	62.14 ± 10.65 ^b^	9.86 ± 1.77 ^a^
Maris Piper	Untreated	10.97 ± 0.66 ^a^	17.87 ± 2.99 ^a^	66.71 ± 13.05 ^a^	11.06 ± 1.24 ^a^
	400 MPa	11.39 ± 0.03 ^a^	17.69 ± 3.04 ^a^	73.20 ± 9.51 ^a^	11.76 ± 0.45 ^a^
	600 MPa	12.65 ± 3.36 ^a^	18.21 ± 2.56 ^a^	67.74 ± 3.02 ^a^	11.61 ± 1.25 ^a^
Maris Piper (baby)	400 MPa	12.18 ± 5.73 ^a^	19.41 ± 2.98 ^a^	117.70 ± 5.80 ^a^	10.43 ± 3.91 ^a^
	600 MPa	11.40 ± 1.18 ^a^	17.78 ± 1.73 ^a^	65.52 ± 0.02 ^b^	10.77 ± 1.09 ^a^
Rooster	Untreated	12.38 ± 2.76 ^a^	16.38 ± 3.17 ^a^	81.60 ± 5.36 ^a^	12.31 ± 2.65 ^a^
	400 MPa	12.23 ± 1.60 ^a^	17.30 ± 1.77 ^a^	78.74 ± 11.50 ^a^	12.32 ± 1.18 ^a^
	600 MPa	12.04 ± 0.84 ^a^	20.97 ± 3.36 ^a^	62.03 ± 8.96 ^b^	11.75 ± 0.75 ^a^

All data are the means ± SD (n = 6). Values with different letters within the same column and variety are statistical different (*p* < 0.05).

## Supplementary Materials

The following is available online at https://www.mdpi.com/article/10.3390/foods10102425/s1. Figure S1: The temperature and pressure profile at 600 MPa.
