# Supplementary material for: High-Pressure Processing on Whole and Peeled Potatoes: Influence on Polyphenol Oxidase, Antioxidants, and Glycaemic Indices
Source: Foods. 2021 Oct 13;10(10):2425. doi: 10.3390/foods10102425 (PMC8535207; doi:10.3390/foods10102425)
Supplement: Supplementary file 1 [file foods-10-02425-s001.zip › foods-1330833-supplementary.pdf]

foods

MDPI

High pressure processing on whole and peeled potatoes: influence on polyphenol oxidase, antioxidants and glycaemic indices

Konstantina Tsikrika[1], Aine Muldoon[2], Nora M. O'Brien[2] and Dilip K. Rai[1]*

[1]Department of Food BioSciences, Teagasc Food Research Centre Ashtown, Dublin5, D15 DY05, Ireland;
[2]School of Food and Nutritional Sciences, University College Cork, Cork, Ireland.
*Correspondence: dilip.rai@teagasc.ie; Tel.: +353 (0)18059569

Supplementary Materials

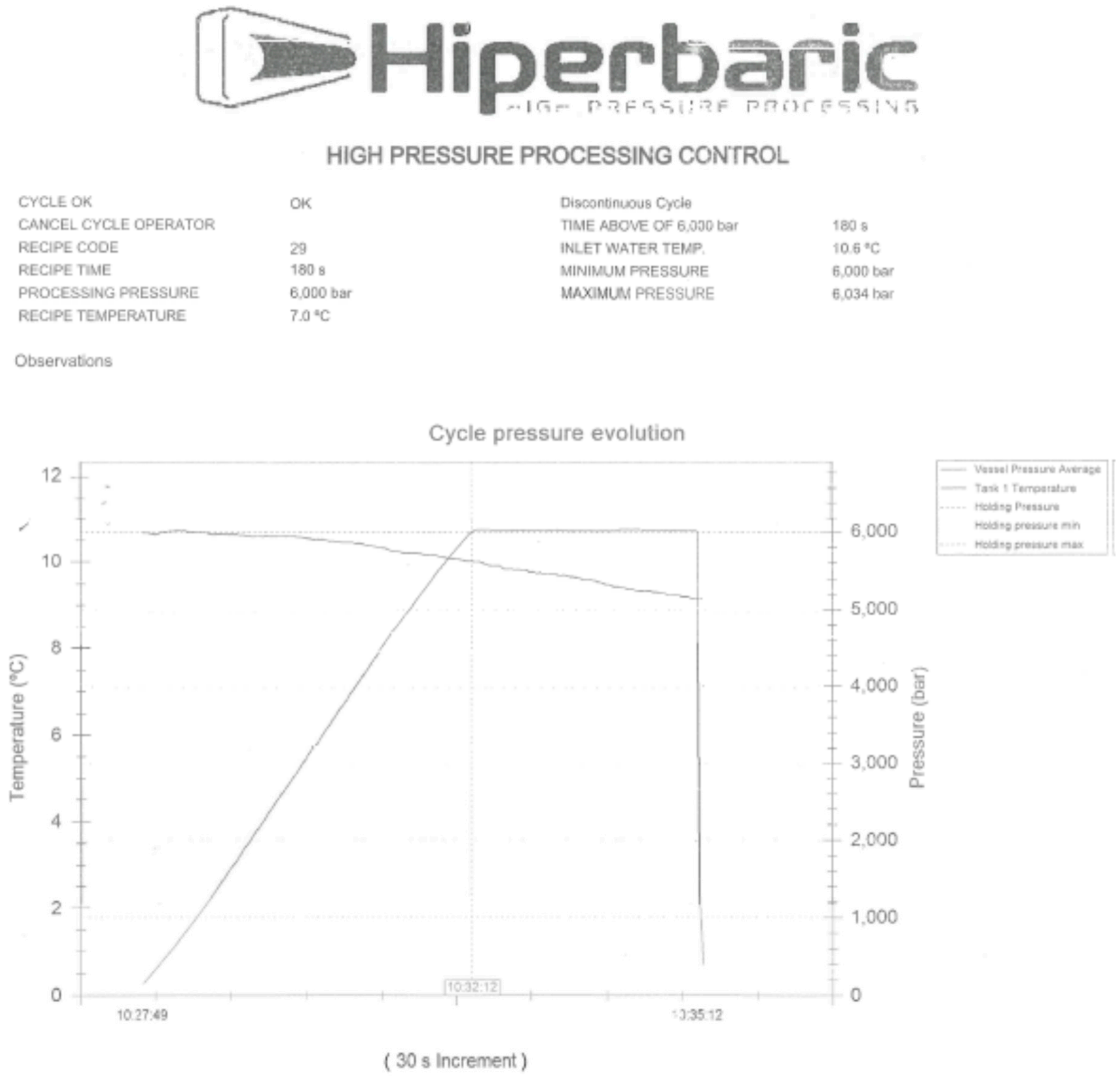

Figure S1: The temperature and pressure profile at 600 MPa.
